# Stewardship of Antibiotics Prescribing in Belgian Dental Practice: A National Survey

**DOI:** 10.3390/ijerph23030282

**Published:** 2026-02-25

**Authors:** Joana C. Carvalho, Dominique Declerck, Peter Bottenberg, Wolfgang Jacquet

**Affiliations:** 1 Institute of Dentistry and Stomatology, Faculty of Medicine and Dentistry, UCLouvain. Av. Hippocrate 10, 1200 Brussels, Belgium; 2 Research Group Population Studies in Oral Health, KU Leuven Department Oral Health Sciences, KU Leuven, Kapucijnenvoer 7, 3000 Leuven, Belgium; dominique.declerck@uzleuven.be; 3 Faculty of Medicine, Université Libre de Bruxelles (ULB), Route de Lennik 808, 1070 Brussels, Belgium; peter.bottenberg@ulb.be; 4 Clinical Sciences (KLIM), Faculty of Medicine and Pharmacy, Vrije Universiteit Brussel (VUB), Laarbeeklaan 103, 1090 Brussels, Belgium; wolfgang.jacquet@vub.be

**Keywords:** antibiotics, prophylaxis, therapeutics, prescription, stewardship, antimicrobial resistance

## Abstract

**Highlights:**

**Public health relevance—How does this work relate to a public health issue?**

- Antibiotics are the most important triggers of antimicrobial resistance.
- According to the World Health Organization, antibiotic resistance is rising to dangerously high levels in all parts of the world.

**Public health significance—Why is this work of significance to public health?**

- This national survey forms part of national actions to contain antimicrobial resistance.
- We examined factors influencing dentists’ prudent antibiotic prescribing, identified barriers to implementing evidence-based guidelines, and proposed strategies to strengthen antimicrobial stewardship in dentistry.

**Public health implications—What are the key implications or messages for practitioners, policy makers and/or researchers in public health?**

- Antibiotics continue to be prescribed for inappropriate indications in certain clinical situations. Continued efforts are needed to raise awareness and strengthen antibiotic stewardship in daily practice.
- Valuable insights into dentists’ antibiotic prescribing practices are offered, supporting targeted interventions to reduce antimicrobial resistance.

**Abstract:**

This national survey examined factors influencing dentists’ prudent antibiotic prescribing, barriers to implementing evidence-based guidelines in dental practice, and solutions to promote prudent prescribing. A validated online survey assessed dentists across five domains: participant characteristics, antibiotic prescribing practices, knowledge of antimicrobial resistance, barriers and solutions for prudent antibiotic use, and knowledge and adherence to evidence-based guidelines. A total of 811 dentists completed the survey (55.2% female). Antibiotic prescribing was mainly influenced by patients’ clinical signs and symptoms (79.0%), immune status (73.0%), and medical/dental history (66.0%). Key contributors to antimicrobial resistance were frequent antibiotic prescribing (97.1%), patient self-medication with leftover antibiotics (95.1%), and use of broad-spectrum agents when narrower options were available (90.3%). Only 25% of participants were familiar with evidence-based guidelines. Regression analysis showed region and professional experience as significantly associated with compliance with prophylactic/therapeutic antibiotic prescribing to protect patients (OR = 1.7–1.5; *p* < 0.025). Awareness of the evidence-based guidelines, working ≤ 30 h/week, and receiving prescription feedback were significantly associated with compliance with therapeutic antibiotic prescribing to protect society (OR = 1.8–1.5; *p* < 0.030). Information technology support was perceived as a useful aid for prescribing. Antibiotics are still prescribed for inappropriate indications. Continued efforts are needed to raise awareness and strengthen antibiotic stewardship in daily practice.

## 1. Introduction

The stewardship of antibiotic prescription in dental practice has gained increasing interest in public health, as antibiotics are the most important triggers of antimicrobial resistance. According to the World Health Organization, antibiotic resistance is rising to dangerously high levels in all parts of the world [1]. Dentists are considered major prescribers of oral antibiotics accounting for 10% of all antibiotic prescriptions in humans [2,3,4]. Nevertheless, current evidence shows that the prescription of antibiotics in dentistry should be considered only in a limited number of clinical conditions and in accordance with evidence-based guidelines [3,5].

A recent systematic review evaluating global trends in antibiotic prescribing for apical periodontitis found that overprescription remains a significant concern. The authors highlighted the need to improve prescribing practices and emphasized the importance of educational initiatives to promote prudent and evidence-based antibiotic use [6]. In this context, several strategies, including audits, education, and digital decision-support tools, have been evaluated for their ability to reduce inappropriate antibiotic prescribing by dentists and strengthen antimicrobial stewardship in dentistry. On average, these interventions resulted in a 70% reduction in inappropriate antibiotic prescriptions, with the largest effects seen in audit-and-education and audit-and-feedback approaches [7].

In the framework of the WHO global action plan on antimicrobial resistance, it is expected that individual countries ensure the implementation of national plans and strategies to contain antimicrobial resistance [1]. While the implementation of nationwide interventions for this purpose becomes urgent, there remains a great need to understand clinical and non-clinical prescribers’ factors contributing to antimicrobial resistance in contemporary populations. According to Zhuo et al. [8], understanding of factors perceived as barriers to the prudent prescription of antibiotics and of strategies to improve prescription practices are equally warranted. Of relevance is the identification of factors considered amenable to changes and potentially leading to a reduction in unnecessary prescriptions.

Since 1999, several initiatives have been taken to improve the prudent prescription and use of antibiotics in Belgium for the human and veterinary sectors [9,10,11,12,13,14]. Recently, the Belgian Health Care Knowledge Centre developed an evidence-based guideline on the prudent prescription of antibiotics in dentistry with clear directives on indications, preferred antibacterial agents, dosage, and duration of use [5]. The extent to which this guideline has been implemented in dental practice and its impact on the mitigation of unnecessary prescriptions are yet to be assessed.

This study examined factors influencing dentists’ prudent antibiotic prescribing, including their prescribing practices, knowledge, and management of antimicrobial resistance. Moreover, it identified key barriers to implementing evidence-based guidelines for antibiotic use in dental care and explored attitudes towards interventions to promote prudent prescribing.

## 2. Materials and Methods

### 2.1. Ethics, Study Design and Sample

The study protocol was submitted to the Ethics Committee of the Free University of Brussels, Belgium, and approved under the Belgian register number EC-2022-095. The study was designed as a national cross-sectional online survey which was carried out between 20 April and 26 June 2022. The present survey is reported according to STROBE guidelines for cross-sectional studies [15]. Dentists were asked to accept or decline the invitation by ticking either a box indicating that they had read the informed consent and privacy policy and voluntarily agreed to data collection and processing, or a refusal box that automatically closed the questionnaire.

In Belgium, there were 8637 dentists registered either as a general dentist or specialist at the moment of conducting this survey [11]. An invitation to participate in the survey was sent to all registered dentists by the National Institute for Health and Disability Insurance. In addition, all dentists included in the contact lists of the main dental associations in Belgium were invited by e-mail. Two reminders were subsequently sent to potential participants by the respective associations. It was anticipated to include approximately 10% of all practitioners, representing both the Dutch- and French-speaking language communities.

### 2.2. Participants

The survey participants included general dentists as well as officially registered specialists in periodontology, orthodontics and maxillofacial surgery employed in private practice, hospital environments or in administrative roles.

### 2.3. Development, Validation, and Test–Retest of the Online Survey

The survey was developed based on a literature review, followed by the selection and adaptation of questions from previous studies that explored the knowledge and practices of physicians and dentists regarding antibiotic stewardship [16,17,18,19,20]. In addition, the survey included questions derived from the evidence-based guideline issued by the Belgian Health Care Knowledge Centre concerning the prudent prescription of antibiotics in dental practice [5]. Most questions were closed-ended with Likert-type responses on a 5-point scale, complemented by a limited number of open-ended questions.

The online survey consisted of five domains: (1) characteristics of the sample, (2) prescription practices of antibiotics for the prevention and treatment of (oral) infections, (3) knowledge and current approaches to managing antimicrobial resistance, (4) opportunities for and barriers to the prudent prescription of antibiotics and possible solutions, and (5) knowledge of and compliance with evidence-based guidelines. The online survey was validated for face and content validity by a panel of 3 experts not involved in its development. The wording, relevance and usefulness of the questions were assessed. The experts were also asked to recommend removal or addition of questions according to their expertise. Amendments were made based on experts’ suggestions. No question was ultimately recommended for removal or addition. The final version of the survey is presented in Supplementary Material (Survey S1). The reliability of the survey was tested-retested with an interval of 1 week by 25 dentists yielding a weighted kappa value of 0.60 (95% CI: 0.57–0.62). The online survey used the Qualtrics^xm^ platform, and all data were collected anonymized.

### 2.4. Benchmark

The benchmark represents a predefined consensus standard based on evidence-based guidelines rather than an absolute measure of clinical appropriateness. The authors established this benchmark by formulating a single consensus answer for the survey questions related to the prudent prophylactic and therapeutic prescription of antibiotics in daily practice [5]. After one week, the responses were retested to assess reliability (κ = 1.00, 95% CI:1.00–1.00]. In a first step, the benchmark was used to compare the answers provided by all survey participants. In a second step, it served to assess general dentists’ compliance with evidence-based guidelines on the prudent prescription of antibiotics in dentistry.

### 2.5. Missing Data

Dentists who completed the survey answered all questions as the Qualtrics^xm^ platform was programmed to prevent missing responses. However, dentists could withdraw at any time, having been informed that the data collected up to that point would still be included in the analysis. If no additional responses were recorded after a defined period, the software automatically terminated data collection, and the remaining unanswered questions were considered missing data.

### 2.6. Statistical Analysis

Survey responses were exported in a Microsoft Excel spreadsheet for data checking and cleaning, then transferred to SPSS ™ (IBM SPSS 26.0.0.0 64-bit) for statistical analysis. Descriptive statistics in terms of absolute and relative frequency were used to present the characteristics of the sample. Weighting was applied to adjust for differences in participation levels by gender and geographical region, based on the official data of Belgian practitioners, with coefficients ranging from 0.86 to 1.31. Descriptive data were used from all participants, further statistical analyses were limited to the group of general practitioners due to scarcity of other profiles. Comparisons between proportions were tested using the χ^2^ test for dichotomous variables, while those between two groups were tested using the Mann–Whitney U test. A significant level of 5% was applied. These tests should be considered exploratory since no correction for multiple testing was applied.

Binary stepwise logistic regression analysis was applied to categorized data to identify factors associated with general dentists’ compliance with the benchmark for prudent prescription of antibiotics. Five distinct outcome categories were considered, reflecting patient-centred, societal, and dentist-centred perspectives on antibiotic stewardship.

Regrouping of the Likert scales was performed based on the rationale that neutrality reflected a limited importance. Each outcome category employed predefined cut-off points to determine agreement with the benchmark. A participant’s response was considered as compliant when it agreed with the acceptable range defined by the benchmark on a 5-point Likert scale. Specifically, when the benchmark indicated that prescribing antibiotics was prudent, a score of 4 or 5 (‘mostly’ or ‘always’) was interpreted as compliant. Conversely, when the benchmark indicated that prescribing antibiotics was contra-indicated, a score of 1 or 2 (‘never’ or ‘seldom’) was considered compliant. The outcome categories were as follows:

- Prudent prophylactic and therapeutic prescriptions from the perspective of protecting patients from further harm (outcomes 1 and 2, respectively): Compliance was assessed using six items. A respondent was considered compliant if at least five out of six answers matched the benchmark.
- Prudent therapeutic prescription from the perspective of protecting society from possible antimicrobial resistance (Outcome 3): This category included ten assessment items. Compliance required a minimum of eight out of ten responses agreeing with the benchmark.
- Prudent prophylactic and therapeutic prescriptions from the perspective of protecting both patients and society (Outcome 4): In this comprehensive category, twenty-two items were evaluated. Compliance was defined as eighteen or more matching responses with the benchmark.
- Prescription attitude (Outcome 5): Compliance in this category was assessed based on a combination of three items reflecting adherence to evidence-based guidelines. Specifically, participants were considered compliant if they regarded the guidelines as a positive development, prescribed antibiotics guided by the patient’s clinical signs and symptoms and did not prescribe antibiotics on patient demand or solely for pain relief.

The regressions analyses were limited to the group of general practitioners (n = 775, Table 1) due to the scarcity of other profiles. Incomplete surveys (n = 43) and surveys in which gender was reported as being “other” (n = 2) were excluded. Consequently, 730 participants were included in the regression analysis. A *p*-value of 0.05 or less was considered statistically significant.

## 3. Results

**Figure 1:**
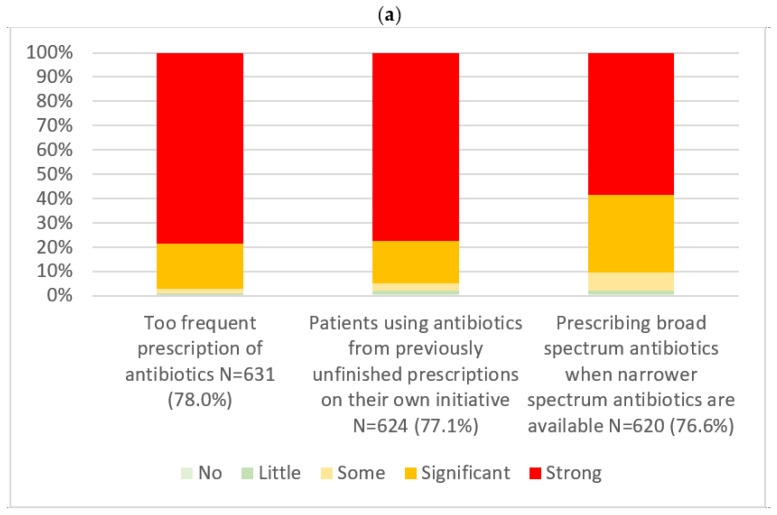

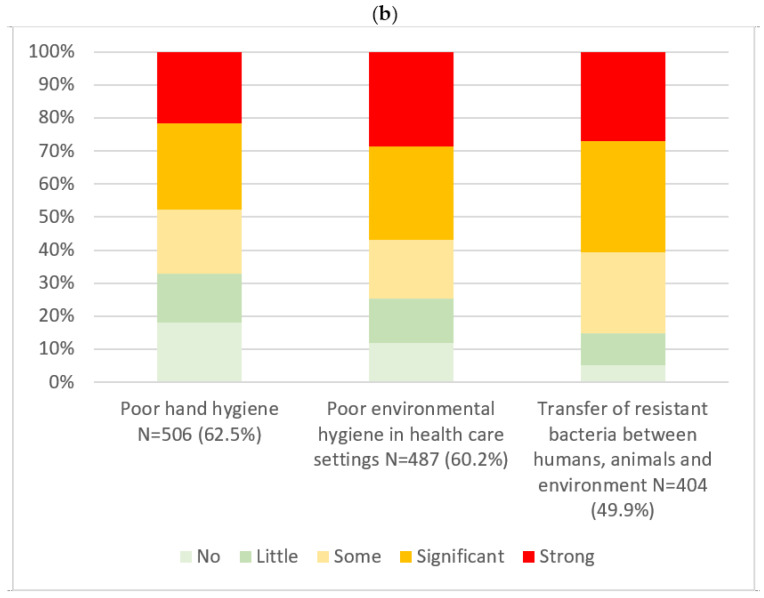
Distribution of participants’ assessments of factors with significant or strong contribution to microbial resistance (**a**) and factors with no or little contribution to microbial resistance (**b**). Responses were given on a Likert scale ranging from no contribution to strong contribution (%). The number of respondents (N) and the corresponding percentages are reported.

**Figure 2:**
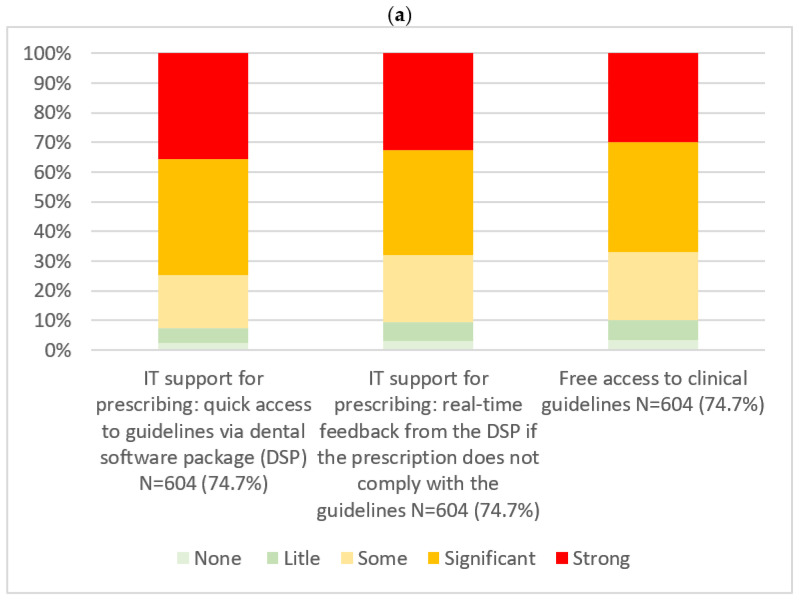

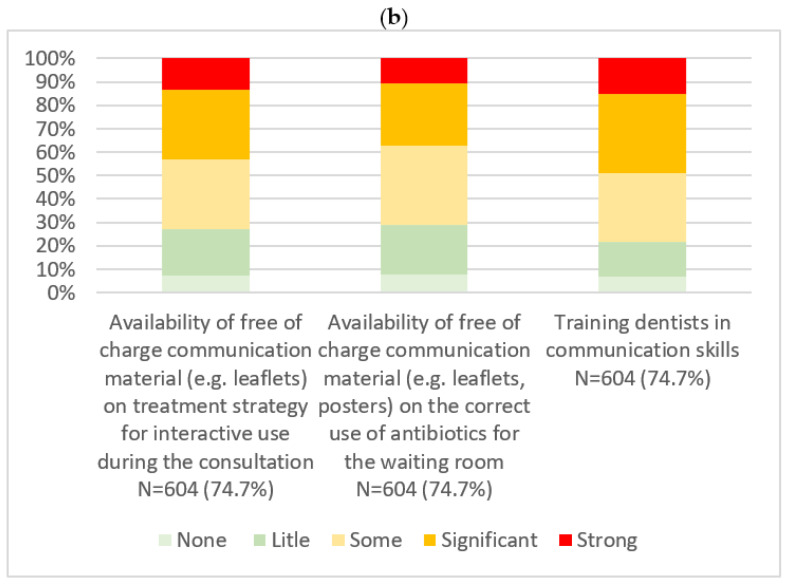
Distribution of participants’ assessments of strategies with significant or strong usefulness for supporting prudent antibiotic prescription (**a**) and strategies with no, little or only some usefulness for the prudent prescription of antibiotics (**b**). Responses were rated on a Likert scale ranging from not useful to very useful (%). The number of respondents (N) and the corresponding percentages are reported.

Among all the participants who answered the online survey (n = 827), a total of 811 participants (98.1%) completed the survey, while 16 (1.9%) refused to participate due to lack of time, retirement, or unusual patient profile. Only 43 general dentists partially completed the questionnaire (5.3%). The median time to complete the survey was 17 min (IQR 12-31). The number of missing answers was calculated and reported for individual variables (Table 1 and Table 2). The descriptive analyses (Table 1 and Figure 1 and Figure 2) included all participants who completed the survey (n = 811). The representativeness of the sample with respect to the Belgian dental workforce population was assessed based on participants’ age, gender, and geographic distribution and was determined by comparing the survey data with those of the Belgian dental workforce [21]. For age and geographic distribution, the Spearman correlation coefficients comparing the characteristics of the study participants with those of the Belgian dental workforce were 0.98 (95% CI: 0.70–0.99, *p* = 0.0040) and 0.86 (95%CI: 0.54–0.96, *p* = 0.0007), respectively, while for gender the χ^2^ test yielded a *p*-value of 0.89.

**Table 1:** Characteristics of the participants (n = 811), number (n) and percentage (%).

Characteristics		n	%
Gender	Female	448	55.2
	Male	361	44.5
	Other	2	0.3
Age	≤34	117	14.4
	35–44	125	15.4
	44–54	188	23.2
	55–64	298	36.7
	≥65	83	10.3
Region *	Brussels-Capital	83	10.2
	Flanders	547	67.4
	Walloon	178	22.0
Professional title **	General dentist	775	95.6
	Periodontist	24	3.0
	Orthodontist	3	0.4
	Maxillo-facial surgeon	2	0.2
Practice ***	Private Solo Practitioner	397	45.6
	Private Group Practitioner	399	45.9
	Hospital	53	6.1
	Academia/Research	21	2.4
	Administration	0	0.0
Professional experience **	<10 years	115	14.2
	11–20 years	144	17.8
	21–30 years	187	23.1
	≥31 years	358	44.1
Working hours per week with patient contact **	<10	11	1.4
	11–20	39	4.8
	21–30	148	18.3
	≥31	604	74.5
	No clinical work	2	0.3
Trainee counsellor **	No	607	74.8
	Yes, currently without trainees	136	16.8
	Yes, currently with trainees	61	7.5
Participation in emergency Service **	No	83	10.2
	Yes	721	88.9

* missing values = 3 (0.4%); ** missing values = 7 (0.9%); *** a participant could have more than one practice

Females represented 55.2% of the sample, the largest age group consisted of participants aged 55–64-year-olds (36.7%), and the majority were general dentists (95.6%). Almost half of participants had more than 30 years of professional experience (44.1%), and about three- quarters treated patients for more than 30 h per week (74.5%). More detailed characteristics of the participants are presented in Table 1.

### 3.1. Prescription Practices of Antibiotics for Prophylaxis and Treatment of (Oral) Infections

Two-thirds of participants (66.2%) reported prescribing antibiotics once a month or less, while only 20 participants (2.5%) reported prescribing them daily. When prescribing antibiotics, most participants indicated that they often or always recorded information in the patient file, including the reason for prescription (76.6%), the type of antibiotic (87.2%), the dosage (80.8%), and the recommended duration of use (71.5%).

In patients with specific health problems, the prudent prophylactic prescription of antibiotics prior to invasive procedures, such as the manipulation of gingival tissue or the periapical region of teeth, perforation of the oral mucosa or tooth extraction, was assessed. Participant agreement with the benchmark reached 87.5% in cases involving patients with a history of infective endocarditis, those with prosthetic heart valve or those who had undergone cardiac valve repair using prosthetic material. However, the agreement with the benchmark was only 31.7% in cases of congenital heart disease without surgical repair. Considerable disagreement with the benchmark was observed, as participants prescribed antibiotics in cases involving diabetic patients with insufficient metabolic control (24.7%), patients with orthopaedic joint replacements (15.5%) and those with poorly controlled high blood pressure (0.3%).

In patients with good general health presenting oral conditions accompanied by systemic involvement, such as fever, facial cellulitis or lymphadenopathy, the therapeutic use of antibiotics may be considered and was reported in specific cases. For dental abscesses in children and adults, 42.0% and 50.0% of the participants prescribed antibiotics, respectively. For patients with apical periodontitis, 44.0% of participants reported prescribing antibiotics, while for those with third molar pericoronitis, the corresponding figure was 46.0%.

In healthy patients presenting conditions without systemic involvement, agreement with the benchmark was observed to some extent: 54.0% of participants reported never prescribing antibiotics in cases of dental extraction, 17.8% of the participants always prescribed antibiotics for dental implants placement, and for irreversible pulpitis, 70.0% and 64.0% of the participants never prescribed antibiotics in children and adults, respectively.

### 3.2. Knowledge and Current Approach of Antimicrobial Resistance

According to participants’ responses, the most important factors significantly or strongly influencing their decision to prescribe antibiotics were a patient’s clinical signs and symptoms (79.0%), a patient’s immune status (73.0%) and a patient’s medical and/or dental history (66.0%). The least important factors, reported as having little or no influence, included the practicing dentists’ workload (58.0%), patients’ expectations (57.0%) and patients’ need for immediate pain relief (42.0%).

Participants reported on the most and least influential factors contributing to antimicrobial resistance, as presented in Figure 1. The factors most identified as significantly or strongly contributing to antimicrobial resistance were the too frequent prescription of antibiotics (97.1%), patients self-medicating with leftover antibiotics from unfinished prescriptions (95.1%), and the use of broad-spectrum antibiotics when narrower-spectrum options are available (90.3%). The factors identified by participants as having no or little contribution to antimicrobial resistance were poor hand hygiene (32.8%), inadequate environmental hygiene in healthcare settings (25.2%), and the transmission of resistant bacteria between humans, animals, and the environment (14.9%).

### 3.3. Opportunities and Barriers to Prudent Prescription of Antibiotics and Possible Solutions

The effectiveness of some measures in supporting the prudent prescription of antibiotics was also assessed based on participants’ responses. Measures rated as effective or very effective were campaigns on the prudent use of antibiotics targeting dentists (45.0%), campaigns targeting the general public (44.0%) and mandatory additional training for dentists on antibiotics prescription and antimicrobial resistance (42.0%). Measures assessed as having little effectiveness or being ineffective were increasing the price of antibiotics for patients (47.0%), imposing financial penalties on dentists who do not prescribe antibiotics in accordance with current evidence-based guidelines (41.0%) and rewarding dentists who demonstrated appropriate antibiotics prescription behavior with accreditation points (32.0%).

The perceived usefulness of strategies to improve antibiotics prescribing, as reported by participants, is detailed in Figure 2. Measures rated as significantly or strongly useful were information technology (IT) support for prescribing with quick access to guidelines via dental software package (DSP) (74.7%), IT support for prescribing providing real-time feedback from the DSP when a prescription does not comply with the guidelines (68.0%), and free access to prescribing guidelines (67.1%). Measures considered to have little or no usefulness included the availability of free communication materials (e.g., leaflets) on treatment strategies for interactive use during the consultations (27.0%), the availability of free communication material (e.g., leaflets, posters) on the correct use of antibiotics for display in waiting rooms (29.0%), and training dentists in communication skills (21.8%).

### 3.4. Knowledge and Compliance with Evidence-Based Guidelines

Only one quarter of the participants reported being familiar with the validated evidence-based guidelines on the prudent prescription of antibiotics issued by the Belgian Health Care Knowledge Centre [5]. Among those who were aware of the guidelines, 14.8% considered them a positive initiative; however, some expressed reluctance to apply the guidelines in daily practice.

In the stepwise logistic regression analyses, none of the independent variables were significantly associated with general dentists’ compliance with the benchmark for prudent antibiotic prescribing across all five outcome categories, which reflected patient-centred, societal, and dentist-centred perspectives on antibiotic stewardship (Table 2). In accordance with the benchmark regarding prophylactic antibiotic prescribing, being located in the Walloon Region (OR = 1.7; 95% CI:1.2–2.5) or the Brussels-Capital Region (OR = 1.9; 95% CI: 1.2–3.0) was significantly associated with prudent prescribing. Dentists with ≤30 years of professional experience were also more likely to prescribe antibiotics prudently (OR = 1.6; 95% CI: 1.2–2.1).

**Table 2:** Stepwise logistic regression of factors associated with compliance of Belgian general dentists with the benchmark regarding prudent antibiotic prescribing outcomes. Number (n); Odds ratio (OR); 95% confidence interval [95% CI]; *p* value; non-significant (ns, *p* > 0.05).

Compliance/ Variables	Prophylactic Prescribing ^a^ n = 299	Therapeutic Prescribing Protecting Patients ^b^ n = 372	Therapeutic Prescribing Protecting Society ^c^ n = 596	Prophylactic & Therapeutic Prescribing ^d^ n = 93	Prescribing Attitude ^e^ n = 508
Sex					
Female (ref.)					
Male	ns	0.6 [0.4–08] *p* < 0.001	ns	ns	ns
Region					
Flandres (ref.)					
Wallonia	1.7 [1.2–2.5] *p* = 0.003	1.5 [1.1–2.2] *p* = 0.025	0.5 [0.3–0.7], *p* < 0.001		
Brussels	1.9 [1.2–3.0] *p* = 0.011	ns	0.5 [0.3–0.0], *p* = 0.004	ns	ns
Experience					
>30 years (ref.)					
≤30 years	1.6 [1.2–2.1) *p* = 0.003	ns	ns	1.7 [1.1–2.6] *p* = 0.021	ns
Trainee counselor					
Yes (ref.)					
No	ns	ns	ns	2.1 [1.3–3.3] *p* = 0.003	ns
Working hours					
>30 h/week (ref.)					
≤30 h/week	ns	ns	1.6 [1.1–2.4] *p* = 0.015	ns	0.6 [0.4–0.9] *p* = 0.005
Prescription					
Weekly (ref.)					
<weekly	ns	1.6 [1.2–2.3] *p* = 0.003	0.7 [0.5–1.0] *p* = 0.026	ns	2.1 [1.5–3.0] *p* < 0.001
Know the guidelines *					
Yes (ref.)					
No	ns	ns	1.8 [1.1–3.0] *p* = 0.012	ns	0.5 [0.3–0.7] *p* < 0.001
Prescription feedback					
Yes (ref.)					
No	ns	ns	1.5 [1.0–2.2] *p* = 0.03	ns	ns
Only private practice					
Yes (ref.)					
No	ns	ns	ns	ns	ns
Emergency service					
Yes (ref.)					
No	ns	ns	ns	ns	ns

* Evidence-based guidelines issued by the Belgian Health Care Knowledge Centre. ^a,b^ Five out of six responses complied with those of the benchmark. ^c^ Eight out of ten responses complied with those of the benchmark. ^d^ Eighteen out of twenty-two responses complied with those of the benchmark. ^e^ All three responses complied with those of the benchmark.

In line with the benchmark regarding therapeutic antibiotics prescribing from the perspective of protecting patients from further harm, being located in the Walloon Region was also significantly associated with prudent prescribing (OR = 1.5; 95% CI:1.1–2.2). In addition, dentists who prescribed antibiotics less than once per week (OR = 1.6; 95% CI: 1.2–2.3) were more likely to prescribe antibiotics prudently. For adherence to the benchmark regarding therapeutic antibiotics prescribing from the perspective of protecting society from possible antimicrobial resistance, significant associations with prudent prescribing were found among dentists who worked ≤ 30 h per week (OR = 1.6; 95% CI: 1.1–2.4), those who reported knowledge of the evidence-based guidelines on antibiotics prescription (OR = 1.8; 95% CI: 1.1–3.0), and those who received feedback on their prescribing patterns (OR = 1.5; 95% CI: 1.0–2.2). For compliance with the benchmark regarding prophylactic and therapeutic antibiotic prescribing, from the perspective of protecting patients from further harm and society from potential antimicrobial resistance, dentists with ≤30 years of professional experience (OR = 1.7; 95% CI: 1.1–2.6) and those who were trainee counsellors (OR = 2.1; 95% CI: 1.3–3.3) were significantly associated with prudent prescribing. Finally, regarding prescribing attitude, only dentists who prescribed antibiotics less than once per week (OR = 2.1; 95% CI: 1.5–3.0) were significantly more likely to prescribe antibiotics prudently.

## 4. Discussion

In dentistry, it is well established that antibiotics are indicated or can be considered in a restricted number of clinical conditions from the perspective of protecting patients from further harm and society from possible antimicrobial resistance [3,5,14,22,23]. The main findings of this national survey were as follows:

- Dentists showed high agreement with the prudent prophylactic prescription of antibiotics before invasive procedures in patients with a history of infective endocarditis, prosthetic heart valves, or cardiac valve repair with prosthetic material. In contrast, considerable disagreement was observed in cases involving antibiotic prescribing for diabetic patients with insufficient metabolic control, patients with orthopaedic joint replacements, and those with poorly controlled hypertension.
- Dentists showed some agreement with the prudent prophylactic antibiotic prescribing practices in cases such as dental extractions and irreversible pulpitis in both children and adults, for which antibiotics are not indicated.
- In patients with good general health but oral conditions with systemic involvement (e.g., fever, facial cellulitis, or lymphadenopathy), in which antibiotics can be considered, dentists recommended therapeutic antibiotics in nearly half of the cases involving dental abscesses, apical periodontitis, and third molar pericoronitis.
- Only one quarter of the dentists reported being aware of the validated Belgian evidence-based guidelines for prudent prescription of antibiotics in daily practice [5].
- Taken together, these findings indicate that although Belgian general dentists demonstrated some adherence to prudent antibiotic prescribing, there is still considerable room for improvement in antibiotic stewardship in daily practice.

Antibiotic stewardship is a set of procedures used to optimize antibiotics prescribing and to ensure effective treatment, minimize adverse effects, prevent antimicrobial resistance, and preserve antibiotic effectiveness [23,24]. At present, Belgium lacks a formal antibiotic-stewardship programme specifically dedicated to dental practitioners [25], although guidelines and surveillance tools for dental antibiotic prescribing have been introduced [5].

When interpreting the results, it is important to note that the benchmark represents a predefined consensus standard derived from evidence-based guidelines and does not constitute an absolute measure of clinical appropriateness. Regarding dentists’ prudent antibiotic prescribing practices for the prophylaxis and treatment of (oral) infections, this survey found that even though dentists expressed concerns about antimicrobial resistance, some of their prophylactic and therapeutic prescribing behaviours may still contribute to its development. This was particularly evident in the prophylactic use of antibiotics prior to invasive procedures in non-cardiac patients as previously observed by Mansour et al. [19]. In this context, it is worth noting that an earlier Belgian study [26] found that antibiotics were prescribed to 4.3% of patients diagnosed with pulpitis. In the present survey, however, 30% of dentists reported prescribing antibiotics to children and 36% to adults in cases of irreversible pulpitis, despite antibiotics being not indicated in such cases. Antibiotic over-prescribing for irreversible pulpitis and apical periodontitis has been reported in other countries [6,27]. The European Academy of Paediatric Dentistry recently published a policy document [28] recommending against routine antibiotic use for localized pulpitis or abscesses without systemic involvement and supporting targeted use only for odontogenic infections with systemic signs.

Furthermore, medical practitioners, dentists and veterinarians understand and are likely receptive to a One Health policy approach to antimicrobial resistance [8]. In our study, dentists’ knowledge and current understanding of antimicrobial resistance appeared limited and somewhat contradictory, which can present barriers to antimicrobial stewardship. On the one hand, over 90% acknowledged that the most influential contributors to antimicrobial resistance included overprescription of antibiotics, patients self-medicating with leftover antibiotics from incomplete courses, and the use of broad-spectrum antibiotics when narrower-spectrum options are available. On the other hand, factors such as poor hand hygiene, inadequate environmental hygiene in healthcare settings, and the transmission of resistant bacteria between humans, animals, and the environment were factors identified by 14.9% to 38.2% of the dentists as having little, or no contribution to antimicrobial resistance. This is reason for concern, as these factors significantly facilitate the spread of resistant bacteria and contribute to the persistence and dissemination of antimicrobial resistance, as well as the development of complex antimicrobial resistance ecosystem [29,30].

With a view to identifying barriers and opportunities to prudent antibiotic prescribing, as well as potential solutions, dentists assessed the usefulness of various tools intended to support this goal. Significant usefulness was attributed to information technology support for prescribing, particularly when it enabled quick access to guidelines via dental software packages. Similarly, information technology support that provided real-time feedback, alerting the prescriber when a prescription did not comply with existing guidelines, was also considered highly useful in accordance with findings of a recent systematic review [7]. In addition, free and unrestricted access to up-to-date prescribing guidelines was rated favourably. Overall, the perceived usefulness of these tools ranged from 67% to 75% among respondents, indicating an opportunity to support dentists in their antibiotic prescribing practices, an approach that aligns with the growing informatization of dental offices in Belgium and many other countries [31,32,33].

A systematic review found that dental antibiotic-prescribing recommendations across clinical practice guidelines varied widely in methodological quality. The authors emphasized the need for greater evidentiary rigour, clearer presentation of information, and improved applicability to enhance implementation and usability [34]. Awareness refers to knowing that something exists, familiarity refers to understanding how it functions and how to apply it, and active use refers to its practical implementation. The lack of awareness does not necessarily indicate resistance to evidence-based practice but may instead reflect issues with dissemination or accessibility. In our study, only one quarter of the dentists reported being aware of the evidence-based guidelines available to them through the Belgian Health Care Knowledge Centre; however, this information was not objectively verified. This finding was surprising given the considerable efforts made to disseminate these guidelines among Belgian dentists through various communication channels. It also helps explain dentists limited adherence to, and reluctance toward implementing the guidelines. Furthermore, only a limited number of practitioners consulted their personal prescription profile available online at the Belgian National Institute for Health and Disability Insurance (NIHDI) webpage.

In 2024, the NIHDI published a report analysing antibiotic prescribing by dentists, examining the distribution and trends of antibiotic consumption in Belgium through reimbursement data of insured individuals from 2013 to 2022 [11]. During this period, 92% of potential dentist prescribers prescribed antibiotics to 3.9% of the insured population, resulting in an expenditure of €3,785,729. The average annual growth rate of antibiotic prescriptions was 0.1%, indicating stability over time. While the overall rate of dentists’ antibiotic prescriptions has remained stable, this survey shows that antibiotics are still prescribed for inappropriate indications in some clinical situations as previously reported in the literature [3,24].

Some limitations of this survey should be acknowledged, particularly the relatively small number of participants (n = 811), who represent 9.4% of the dentist workforce in Belgium, which limits the generalizability of the findings. However, the representativeness of the sample was assessed, and data weighting was applied to enhance the generalizability of the results. The potential for non-response bias, particularly regarding dentists less engaged in antibiotics stewardship initiatives, the possibility that participants represent a more stewardship-aware subgroup, and the potential subjectivity inherent in translating guideline recommendations into Likert-scale cut-offs are acknowledged. Furthermore, this survey focused on whether antibiotics were indicated or not, with particular emphasis on a better understanding of the factors influencing dentists’ prescription decisions in their daily practice as recommended by Thompson et al. [3]. While correct indication is important, the molecule, dose, duration, and overall quality of the prescription are equally crucial; however, these aspects were not assessed in this study. Lastly, the cross-sectional design of the study allows for the identification of associations between factors related to dentists and the prudent prescription of antibiotics but does not permit any inference of causality.

Despite these limitations, this study also has strengths that enhance the validity and relevance of its findings. One of these is the use of an online survey validated by a panel of experts who were not involved in its development. In addition, the reliability of the survey was assessed through a test–retest procedure with dentists. Another strength is the use of a benchmark to measure general dentists’ compliance with evidence-based guidelines on the prudent prescription of antibiotics in dentistry. Finally, the survey was designed to generate data relevant to policy development, both locally and in other countries seeking to improve antibiotic stewardship in daily dental practice.

## 5. Conclusions

In conclusion, the findings of this survey indicate that, although Belgian general dentists demonstrated some adherence to prudent antibiotic prescribing guidelines, antibiotics are still prescribed for inappropriate indications in clinical situations. There remains considerable room for improvement in antibiotic stewardship in daily practice. Continued efforts are needed to raise awareness of antimicrobial resistance and promote the prudent use of antibiotics among Belgian dentists, as only one quarter of them reported being aware of the evidence-based guidelines available to them through the Belgian Health Care Knowledge Centre.

## Data Availability

The data supporting the results of this study are available in a public repository: https://figshare.com/ (accessed on 3 December 2025).

## Supplementary Materials

The following supporting information can be downloaded at https://www.mdpi.com/article/10.3390/ijerph23030282/s1. Survey questionnaire Stewardship of Antibiotics Prescribing in Belgian Dental Practice: A National Survey, Survey questionnaire
