# Supplementary material for: Stewardship of Antibiotics Prescribing in Belgian Dental Practice: A National Survey
Source: Int J Environ Res Public Health. 2026 Feb 25;23(3):282. doi: 10.3390/ijerph23030282 (PMC13027060; doi:10.3390/ijerph23030282)
Supplement: Supplementary file 1 [file ijerph-23-00282-s001.zip › ijerph-4110008-supplementary.pdf]

Stewardship of Antibiotics Prescribing in Belgian Dental Practice: A National Survey

Survey questionnaire

1. General information

Gender ☐ Female ☐ Male ☐ Other

Age group

Postal code of your main dental workplace

Do you agree to participate?

☐ Yes No ☐

Reason for not answering this questionnaire:

Reason for refusing: ☐ No interest
☐ No time
☐ Other, specify ………………………………………………………..

(For those refusing participation the questionnaire terminates here)

2. Information on the participating dentist

ID.01. Are you a:
(tick one only)

General dentist (including practice related to a specific discipline) .... ☐

Specialist in orthodontics .... ☐

Specialist in periodontics .... ☐

Specialist in maxillo-facial surgery.. ☐

ID.02.Please describe your current dental workplace
(tick all that apply)

Private practice solo .... ☐

Private practice group .... ☐

Hospital practice.. ☐

Academic/research .... ☐

Administration.. ☐

ID.03. How many years have you been working as a dentist?
(tick one only)

≤10.. ☐

11-20 .... ☐

21-30 .... ☐

≥31.. ☐

ID.04.How many hours per week do you provide chairside patient care?
(not including administration) (tick one only)

< 10.. ☐

10-20 .... ☐

21-30 .... ☐

≥31.. ☐

Not applicable, my work is non-clinical.. ☐

ID.05.Are you a trainee counselor?

(tick only one)

No, I am not.. ☐

Yes, I am but I currently have no trainee .... ☐

Yes, I am and I do have currently a trainee .... ☐

ID.06.Are you actively participating in emergency service/care?
(actually delivering patient care)

No .... ☐

Yes .... ☐

3. Prescription practices of antibiotics for prophylaxis and treatment of local or general infection

---

AP.01. For which of the following cases/conditions would you consider PROPHYLACTIC use of antibiotics prior to an invasive dental intervention:
(manipulations of the gingival tissue, periapical region of teeth or perforation of the oral mucosa, dental extraction) (please tick all that apply)

Diabetes with insufficient metabolic control .... ☐

Orthopaedic joint implants.. ☐

History of infective endocarditis.. ☐

Poorly controlled high blood pressure .... ☐

Congenital heart disease without surgical repair .... ☐

Prosthetic valve or a prosthetic material used for cardiac valve repair................ ☐

AT.01. How often do you prescribe antibiotics for TREATMENT of oral infections?
(tick one only)

Daily ☐
Weekly ☐
Monthly ☐
Less often than monthly ☐
I do not prescribe antibiotics ☐ (conditional jump to PP.05.)

AT.02. For which of the following conditions would you consider prescribing antibiotics when <u>TREATING</u> patients in good general health:
(please tick one in each row)

	Never	Rarely	Sometimes	Often	Always
Symptomatic irreversible pulpitis in the primary dentition	○	○	○	○	○
Symptomatic irreversible pulpitis in the permanent dentition	○	○	○	○	○
Odontogenic abscess in the primary dentition with systemic involvement (*e.g. fever, facial cellulitis, lymphadenopathy*)	○	○	○	○	○
Odontogenic abscess in the primary dentition without systemic involvement	○	○	○	○	○
Symptomatic apical periodontitis in the permanent dentition with systemic involvement *(e.g. fever, facial cellulitis, lymphadenopathy)*	○	○	○	○	○
Symptomatic apical periodontitis in the permanent dentition without systemic involvement	○	○	○	○	○
Acute apical abscess in the permanent dentition with systemic involvement *(e.g. fever, facial cellulitis, lymphadenopathy)*	○	○	○	○	○
Acute apical abscess in the permanent dentition without systemic involvement	○	○	○	○	○
Replantation of avulsed permanent teeth	○	○	○	○	○
Non-surgical treatment of aggressive periodontitis	○	○	○	○	○

Periodontal abscess in the permanent dentition with systemic involvement *(e.g. fever, facial cellulitis, lymphadenopathy)*	○	○	○	○	○
Periodontal abscess in the permanent dentition without systemic involvement	○	○	○	○	○
Pericoronitis of third molars with systemic involvement *(e.g. fever, facial cellulitis, lymphadenopathy)*	○	○	○	○	○
Pericoronitis of third molars without systemic involvement	○	○	○	○	○
Extraction of permanent teeth	○	○	○	○	○
Dental implant placement	○	○	○	○	○

AT.03. If you prescribe antibiotics, do you record the following information in the patient's file? (please tick one for each row)

Reason for antibiotic prescription: Never ☐ Rarely ☐ Sometimes ☐ Often ☐ Always ☐

Type of antibiotic: Never ☐ Rarely ☐ Sometimes ☐ Often ☐ Always ☐

Dosage of antibiotic: Never ☐ Rarely ☐ Sometimes ☐ Often ☐ Always ☐

Advised duration of use: Never ☐ Rarely ☐ Sometimes ☐ Often ☐ Always ☐

4. Factors influencing a prudent prescription of antibiotics

PP.01. How important do you rate each of the following sources of knowledge on the prudent prescription of antibiotics?
(please tick one for each row)

	Not important	Little importance	Some importance	Important	Very important	I don't know
Dental school training	○	○	○	○	○	○
Clinical Practice Guidelines	○	○	○	○	○	○

(e.g. BAPCOC, KCE, ...)						
Advice of other dentists	○	○	○	○	○	○
Advice of medical doctors	○	○	○	○	○	○
Scientific literature	○	○	○	○	○	○
Periodicals issued by dental associations	○	○	○	○	○	○
Internet resources	○	○	○	○	○	○
Continuing education	○	○	○	○	○	○
Previous clinical experience	○	○	○	○	○	○

PP.02. Which factors influence your decision whether or not to prescribe antibiotics?

(please tick one for each row)

	No influence	Little Influence	Some influence	Influence	Strong influence
Patient's clinical signs/symptoms	○	○	○	○	○
Patient immune status	○	○	○	○	○
Patient's medical and/or dental history	○	○	○	○	○
Patient's history of antibiotic use	○	○	○	○	○
Guideline recommendations	○	○	○	○	○
My experience in managing similar problems	○	○	○	○	○
Patient safety	○	○	○	○	○
Risk of promoting antimicrobial resistance in the patient	○	○	○	○	○
Potential adverse/side effects in taking antibiotics	○	○	○	○	○
Community wide-risk of antimicrobial resistance	○	○	○	○	○
Immediate pain relief	○	○	○	○	○
Patient expectation	○	○	○	○	○
Workload	○	○	○	○	○

Feasibility of delivering operative dental procedures (technical reason or lack of cooperation)	○	○	○	○	○

PP.03. Which guidelines for the prescription of antibiotics in dental patients do you know and apply?

(please tick all that apply in each column)

	Know	Apply
BAPCOC (Belgian Antibiotic Policy Coordinator Committee) (conditional jump: go to PP.05.)	☐	☐
KCE (Belgian Health Care Knowledge Centre) (conditional jump: go to PP.05.)	☐	☐
CEBAM (Belgian Center for Evidence-based Medecine) (conditional jump: go to PP.05.)	☐	☐
Guidelines from the hospital I work (conditional jump: go to PP.05.)	☐	☐
BCFi/CBiP guide (conditional jump: go to PP.05.)	☐	☐
International (non-Belgian) guidelines (conditional jump: go to PP.05.)	☐	☐
Other, specify: (conditional jump: go to PP.05.)	☐	☐
I do not know/apply guidelines (conditional jump: go to PP.04.)	☐	☐

PP.04. Reasons for not using guidelines to support your decision to prescribe antibiotics:

(check all that apply)

- Lack of time to keep up with guidelines and evidence ☐
- Conflicting guidelines and practices between professions (e.g. between dentists and physicians) ☐
- Conflicting scientific evidence ☐
- Lack of information on antibiotic selection ☐
- Feeling confident that my antibiotics prescription is correct ☐
- Available guidelines are impractical, too complex, difficult to consult.. ☐
- Other, specify ☐

PP.05. To what extent do you agree with the following statements?

(tick one in each row)

	Strongly disagree	Disagree	Neutral	Agree	Strongly agree	I don't know
Antimicrobial resistance will become a greater clinical problem in the future than it is today	○	○	○	○	○	○

In recent years I have become more aware of antimicrobial resistance	○	○	○	○	○	○
Antibiotic use in a patient may reduce its effectivity in the same individual in the future	○	○	○	○	○	○
Antibiotic use in a patient may reduce its effectivity for other individuals in the future	○	○	○	○	○	○
The antibiotic that I prescribe contributes to the problem of antimicrobial resistance	○	○	○	○	○	○
A single course of antibiotic can cause antimicrobial resistance	○	○	○	○	○	○
Antimicrobial resistance can last a year in a patient after a single dose of antibiotics	○	○	○	○	○	○
New antibiotics will be developed that will keep with up the problem of antimicrobial resistance	○	○	○	○	○	○
Dentists can play an important role in reducing antimicrobial resistance	○	○	○	○	○	○
I am interested in playing a role in combating antimicrobial resistance by better self-monitoring and antibiotic stewardship	○	○	○	○	○	○

PP.06. To what extent do you think the following factors contribute to antimicrobial resistance?
(please tick one in each row)

	No contribution	Little contribution	Some contribution	contribution	Strong contribution	I don't know
Antibiotic use in minor or self-limited illnesses	○	○	○	○	○	○
Too frequent antibiotics prescription	○	○	○	○	○	○
Prescribing broad spectrum antibiotics when narrower spectrum antibiotics are available	○	○	○	○	○	○
Prescribing an antibiotic when benefit to patient is uncertain	○	○	○	○	○	○
Prescribing antibiotics without removing the source of infection	○	○	○	○	○	○

Patients using antibiotics from previously unfinished prescription on their own initiative	○	○	○	○	○	○
Patients not finishing the prescribed course of antibiotic	○	○	○	○	○	○
Too low doses of antibiotics	○	○	○	○	○	○
Too short duration of antibiotic treatment	○	○	○	○	○	○
Too long duration of antibiotic treatment	○	○	○	○	○	○
Transfer of resistant bacteria between humans, animals and environment	○	○	○	○	○	○
Environmental contamination with antibiotic waste (farms, hospitals, homes)	○	○	○	○	○	○
Poor environmental hygiene in health care settings	○	○	○	○	○	○
Poor hand hygiene	○	○	○	○	○	○

PP.07. To what extent are you confident that the following organizations take policy action to combat antimicrobial resistance?
(tick one only in each row)

	No confidence	Little confidence	Some confidence	Confident	Very confident
Governmental agencies (INAMI /RIZIV, FAGG/AFMPS, BAPCOC, KCE/CFESS)	○	○	○	○	○
EBPracticenet	○	○	○	○	○
Dental Professional Associations	○	○	○	○	○
Local study clubs	○	○	○	○	○
Locoregional health platform	○	○	○	○	○
Academia	○	○	○	○	○

SS.01. How effective do you think the following measures would be in combating antimicrobial resistance?
(tick one only in each row)

	Not effective	Little effectiveness	Some effectiveness	Effective	Very effective
Restricting the prescription of certain antibiotics	○	○	○	○	○
Restricting the prescription by dentists of certain antibiotics	○	○	○	○	○
Increasing the price of antibiotics for patients	○	○	○	○	○
Increase the accessibility of emergency dental care	○	○	○	○	○
Timely verification of antibiotic prescribing behavior (audit and feedback) by an external party	○	○	○	○	○
Rewarding dentists with correct antibiotic prescribing behavior with accreditation points	○	○	○	○	○
Financial penalties for dentists with poor quality antibiotic prescribing behavior	○	○	○	○	○
Mandatory information on the indication (diagnosis) of each antibiotic prescription	○	○	○	○	○
Oral health awareness campaign on prevention of dental infection	○	○	○	○	○
Campaign on the prudent use of antibiotics aiming at the dentists	○	○	○	○	○
Campaign on the prudent use of antibiotics aiming at the public	○	○	○	○	○
Mandatory additional training on antibiotic prescribing and antimicrobial resistance for dentists	○	○	○	○	○
Enabling patients to obtain the exact number of prescribed antibiotic tablets instead of a whole package	○	○	○	○	○

SS.02. In your opinion, what would be the usefulness of the following elements in improving antibiotics prescription?
(tick one in each row)

	Not useful	Little usefulness	Some usefulness	useful	Very useful
IT support for prescribing: quick access to guidelines via dentist software package (DSP)	○	○	○	○	○
IT support for prescribing: real-time feedback from the DSP if the prescription does not comply with the guidelines	○	○	○	○	○
Training of dentists: communication skills	○	○	○	○	○
Training of dentists: how to apply clinical guidelines in daily practice	○	○	○	○	○
Training of dentists: understanding antimicrobial resistance	○	○	○	○	○
Training of dentists: decision-making skills	○	○	○	○	○
Availability of free of charge communication material (e.g. leaflets, posters) on the correct use of antibiotics for the waiting room	○	○	○	○	○
Availability of free of charge communication material (e.g. leaflets) on treatment strategy for interactive use during the consultation	○	○	○	○	○
Free access to clinical guidelines	○	○	○	○	○
Real-time access to indicators of the quality of your antibiotics prescribing behavior via your DSP for self-assessment	○	○	○	○	○
Real-time access to indicators of the quality of your antibiotics prescribing behavior via INAMI/RIZIV for self-assessment	○	○	○	○	○

SS.03. Do you use patient focused strategies to highlight the importance of the prudent use of antibiotics?
(tick all that apply)

I ensure that information material is available in the waiting room ………………... ☐
I hand out the information material to patients……………………………………… ☐
I discuss interactively the information material with the patients…………………... ☐
I discuss informally with my patients…………………. ....……………….. ☐
I do not advocate patient focused strategies…………… ....……………… ☐
Other, specify……………………………………………………………………….... ☐

SS.04. What form of training would you prefer to keep up to date your antibiotics prescription?
(please tick all that apply)

Reading material .... ☐
Workshop .... ☐
Congress, seminars………….. ☐
Informal training at workplace.. ☐
E-learning.. ☐
Other, specify…………... ☐
I am not interested in further training .... ☐

SS.05. Which institution should provide the training to keep up to date dentist's antibiotics prescription? (please tick all that apply)

Dental associations.. ☐
Universities……………………………………………………... ☐
Other, specify .... ☐

SS.06. Do you know the KCE guideline on the prudent prescription of antibiotics? (If "No" the survey terminates here)

Yes ☐ No ☐

SS.07. What is your opinion on the new KCE guideline on the prudent prescription of antibiotics?

(check only one)

A positive change, I was concerned about the risk of antimicrobial resistance………….. ☐
A too radical change, I do not totally agree with it………………………..................... ☐
I would like to discuss it with my peers before I would be willing to implement changes. ☐
I would like to get further information before I would be willing to implement changes... ☐
Other, specify………………………………………………………………………….. ☐

SS.08. Do you intend to implement the new KCE guideline on the prudent prescription of antibiotics?

(check only one)

Yes, I am already applying it……. ....……………☐
Yes, I will be implementing these changes from now on……………………….................☐
Not yet, but I am considering to implement them in my clinical work………………………..☐
No, I will request guidance from professional associations before implementing it............ ☐
No, I will request guidance from universities before implementing it. .... ☐

No, I do not agree with these changes and will not be implementing the new KCE guideline. ☐

Other, specify……………………………………………………………………………… ☐

Thank you for your cooperation!
